# Phlorofucofuroeckol-A: A Natural Compound with Potential to Attenuate Inflammatory Diseases Caused by Airborne Fine Dust

**DOI:** 10.3390/medicina61010165

**Published:** 2025-01-20

**Authors:** Eun-Gyeong Lee, Sung-Kun Yim, Sang-Min Kang, Byung Jae Ahn, Chang-Kwon Kim, Mina Lee, Dongseob Tark, Gun-Hee Lee

**Affiliations:** 1 Laboratory for Infection Disease Prevention, Korea Zoonosis Research Institute, Jeonbuk National University, Iksan 54531, Republic of Korea; eunlee@jbnu.ac.kr (E.-G.L.); sangminkang@jbnu.ac.kr (S.-M.K.); 2 Marine Biotechnology Research Center, Jeonnam Bioindustry Foundation, Wando-gun 59108, Republic of Korea; skyim0619@gmail.com (S.-K.Y.); bjahn@jbf.kr (B.J.A.); 3 College of Pharmacy and Research Institute of Life and Pharmaceutical Sciences, Sunchon National University, Suncheon 57922, Republic of Korea; ckkim2149@scnu.ac.kr (C.-K.K.); minalee@scnu.ac.kr (M.L.)

**Keywords:** fine dust, PFF-A, inflammation, seaweed extract

## Abstract

*Background and Objectives*: Persistent exposure to airborne fine dust (FD) particles contributing to air pollution has been linked to various human health issues, including respiratory inflammation, allergies, and skin diseases. We aimed to identify potential seaweed anti-inflammatory bioactive reagents and determine their effects on systemic inflammatory responses induced by FD particles. *Materials and Methods*: While exploring anti-inflammatory bioactive reagents, we purified compounds with potential anti-inflammatory effects from the seaweed extracts of *Ecklonia cava*, *Ecklonia stolonifera*, and *Codium fragile*. Structural analyses of the purified compounds siphonaxanthin (Sx), fucoxanthin (Fx), dieckol (Dk), and phlorofucofuroeckol-A (PFF-A) were performed using NMR and LC-MS/MS. *Results*: Notably, these compounds, especially PFF-A, showed significant protective effects against FD-induced inflammatory responses in RAW 264.7 cells without cytotoxicity. Further investigation of inflammatory-associated signaling demonstrated that PFF-A inhibited IL-1β expression by modulating the NF-κB/MAPK signal pathway in FD-induced RAW 264.7 cells. Additionally, gene profiling revealed the early activation of various signature genes involved in the production of inflammatory cytokines and chemokines using gene profiling. Treatment with PFF-A markedly reduced the expression levels of pro-inflammatory and apoptosis-related genes and even elevated the Bmp gene families. *Conclusions*: These results suggested that PFF-A is a potential natural therapeutic candidate for managing FD-induced inflammatory response.

## 1. Introduction

Introduction of high particulate matter (PM) into the air has become a significant issue that causes various environmental and health problems [1,2,3]. PM, classified as an air pollutant comprising suspended particles, is not a single pollutant but a mixture of many chemical species, including organic and inorganic [4,5,6]. In particular, the environmental conditions of air pollution, including PM generated from vehicle emissions and coal combustion, are known to cause allergies and inflammatory and respiratory diseases such as asthma [7,8]. The World Health Organization (WHO) estimates that PM air pollution is the 13th leading cause of mortality worldwide, significantly contributing to several premature deaths annually [9]. Inflammation, the immune response to tissue damage, is triggered by stressful and harmful stimuli, including pathogens, chemicals, irritants, or physical injury, and involves the coordinated communication of various immune cells. This inflammatory response is a common hallmark of the pathogenesis of several diseases associated with the exposure to air pollutants, including PM. Recently, PM exposure has been shown to increase the risk of both pulmonary and systemic inflammatory responses. Although it remains unknown how PM exposure causes inflammatory diseases, a potential mechanism for disease susceptibility may be associated with epigenetic changes [1,10]. Therefore, several previous studies have emphasized the importance of developing potential therapeutic strategies for PM-inflammatory diseases that threaten human health [11,12]. Natural compounds derived from seaweed extracts have recently attracted attention owing to their potent biofunctional properties and nutraceutical benefits [13]. Previous studies have suggested that natural bioactive substances do not have much effect on the human body for a short period but can significantly improve health outcomes through consistent intake [14]. The biological and chemical diversity of seaweeds in marine environments constitutes an important storage of novel bioactive compounds, such as steroids, polysaccharides, alkaloids, fatty acids, and proteins. Some seaweed isolates exhibit several types of anti-inflammatory properties [15]. The carotenoids used in this study, siphonaxanthin (Sx) and fucoxanthin (Fx), have been reported to have effective antioxidant activity, possibly through the excess energy transfer of singlet oxygen in the long central allenic chain [16]. Moreover, allenic bonds and other functional groups may contribute to antioxidant properties by reacting with free radicals [17]. Polyphenols, which produce various derivatives, are found only in brown algae and are reported to have antioxidant and radical-scavenging properties, as well as anticancer and anti-inflammatory effects [18]. Although the exact biological roles of phlorotannins have not been fully elucidated, the phlorotannins used in this study, dieckol (Dk) and phlorofucofuroeckol-A (PFF-A), have been reported to exhibit neuroprotective, anti-allergic, and anti-inflammatory activities [19,20].

Here, we purified four single compounds from seaweed extracts containing brown and green algae using chloroform and ethyl acetate partitioning and subjected them to structural analysis by nuclear magnetic resonance (NMR) and liquid chromatography-mass spectrometry (LC-MS). Next, we investigated whether commercial fine dust (FD), PM10-like, accelerates the inflammatory response in RAW 264.7 cells by analyzing the production of pro-inflammatory cytokines and the activation of inflammatory mediators. Furthermore, we evaluated the potent anti-inflammatory effects of purified single compounds in RAW 264.7 cells stimulated with FD.

## 2. Materials and Methods

### 2.1. Cells, Antibodies, and Materials

RAW 264.7 cells were cultured in Dulbecco’s modified Eagle’s medium (DMEM, Thermo Fisher Scientific, Waltham, MA, USA) containing 10% fetal bovine serum (FBS, Thermo Fisher Scientific, Waltham, MA, USA), 100 U/mL of penicillin, and 100 μg/mL of streptomycin (Thermo Fisher Scientific, Waltham, MA, USA). The antibodies used were monoclonal mouse anti-phospho-NF-κB, p65, anti-NF-κB, p65, anti-phospho-IκB, and anti-IκB; polyclonal rabbit anti-phospho-ERK1/2, anti-ERK1/2, anti-phospho-p38, anti-p38, anti-phospho-JNK, and anti-JNK; and monoclonal mouse anti-IL-1β and anti-β-actin (Cell signaling Technology, Beverly, MA, USA). The secondary antibodies used were horseradish peroxidase-conjugated goat anti-rabbit and horseradish peroxidase-conjugated goat anti-mouse IgG-HRP (Cell Signaling Technology, Danvers, MA, USA). The fine dust (PM10-like) European reference materials ERM-CZ100 (organic constituents), ERM-CZ120 (inorganic constituents), and LPS were purchased from Sigma-Aldrich (St. Louis, MO, USA). The FD particle and LPS were dissolved in DMSO, with each FD particle at a 100 mg/mL stock concentration and LPS at 1 mg/mL. Fucoxanthin standard was purchased from Sigma-Aldrich, and dieckol and phlorofucofuroeckol-A were purchased from Biosynth (Compton, UK). All purchased and purified single compounds were dissolved in DMSO at a 10 mM stock concentration. Analytical-grade organic solvents, including acetonitrile, ethanol, methanol, and deionized water, were purchased from Burdick & Jackson Chemicals (Muskegon, MI, USA).

### 2.2. Purification of a Single Compound from Seaweed Extracts

The algae *Ecklonia stolonifera* and *Codium fragile* were obtained from Wando, Jeollanam-do, South Korea, and dried *Ecklonia cava* was purchased from Jejusea Green (Jeju, Republic of Korea). Fresh seaweed was washed with tap water to remove salt, epiphytes, and sand attached to the surface of the samples and then dried. Dried *E. stolonifera* and *C. fragile* were extracted with 95% ethanol, twice, respectively, and *E. cava* was treated twice with 70% ethanol for 4 h at 70 °C under reflux conditions. Each ethanol extract was filtered through cotton wool and evaporated under reduced pressure. Crude *E. stolonifera* and *C. fragile* extracts were partitioned between water and chloroform to obtain the fucoxanthin (Fx) and siphonaxanthin (Sx) extracts. The *E. cava* extract was suspended in water and partitioned sequentially with an equal volume of hexane, chloroform, and ethyl acetate. The ethyl acetate fraction was evaporated to obtain phlorotannin-containing Dieckol (Dk) and phlorofucofuroeckol-A (PFF-A) extracts. The extracts were dissolved in acetonitrile, filtered using a disposable filter of 0.45 um pore size, and subjected to preparative HPLC using water fraction collector III, Waters, Milford, MA, USA, on an Atlantis prep dC18 OBD column with a C18 guard cartridge (SunFire prep C18, Waters, Milford, MA, USA). Fx and Sx were eluted using an acetonitrile/water mixture at a ratio of 75:25 (*v*/*v*). Dk and PFF-A were eluted with a stepwise gradient of solvents A (water) and B (acetonitrile). The elution gradient was as follows: 0–10 min, 0–10% B; 10–40 min, 10–40% B; 40–55 min, 40–10% B. The flow rate was 10 mL/min, Fx and Sx were detected at 450 nm, and others at 290 nm. Fx, Sx, Dk, and PFF-A were collected and condensed using a rotary evaporator and stored at −80 °C.

### 2.3. Analytic Procedures for Purified Single Compounds

Purified single compounds were analyzed by reverse-phase HPLC using an Arc-HPLC system (Waters, Milford, MA, USA). To separate Fx and Sx, the isocratic mobile phase was acetonitrile, methanol, and 0.1% formic acid (75:15:10, *v*/*v*/*v*) at a flow rate of 1 mL/min. The mobile phase for Dk and PFF-A was acetonitrile-water in gradient mode, which was as follows: water with 0.1% formic acid–acetonitrile with 0.1% formic acid. The chromatographic peaks of Fx, Sx, Dk, and PFF-A were identified by comparing the retention times and spectra of standard samples. NMR spectra were recorded on a JEOL JNM-AL (400 MHz) spectrometer (JEOL, Tokyo, Japan), and chemical shifts were expressed as δ values (ppm) with TMS as the internal standard (measured in methanol-d4). LC-MS/MS analysis was performed using an Orbitrap Exploris 120 mass spectrometer with a Vanquish UHPLC system (Thermo Fisher Scientific, Sunnyvale, CA, USA).

### 2.4. Cytotoxicity Assay

The cytotoxicity of FD and the individual compounds was evaluated using a LUNA II cell counter (Logos Biosystems, Anyang, Republic of Korea) and Cell Titer-Glo Luminescent Cell Viability Assay Kit (Promega, Madison, WI, USA). RAW 264.7 cells were seeded into 96-well plate at a density of 1 × 10^4^ cells/well and incubated for 24 h. The cells were then washed twice with PBS and treated with FD at the indicated concentrations for 24 h. Cell viability was measured using a LUNA II cell counter following trypan blue staining. To assess the cytotoxicity of the purified single compounds, RAW 264.7 cells were seeded into a 96-well plate at a density of 1 × 10^4^ cells/well and incubated for 24 h. The cells were washed twice with PBS and then treated with purified single compounds at experimental concentrations for 24 h. Cell Titer-Glo reagent (2.0) was added to each well, and cell viability was calculated using a GloMax Discover microplate reader (Promega, Madison, WI, USA).

### 2.5. Immunoblotting

RAW 264.7 cells were seeded into a 6-well plate (2 × 10^6^ cells/well) and pretreated with 10 μM of PFF-A for 3 h. After 3 h, FD or LPS was treated at the indicated times. The harvested RAW 264.7 cell lysates were lysed using the RIPA buffer (Thermo Fisher Scientific, Rockford, IL, USA), and the supernatant was collected after centrifugation. The supernatant was mixed with 4× Laemmli sample buffer (Bio-Rad, Hercules, CA, USA), boiled, and subjected to sodium dodecyl sulfate-polyacrylamide gel electrophoresis. The separated proteins were transferred to the PVDF membranes and then blocked with 5% skim milk. Membranes were then incubated with specific antibodies. Protein expression levels were detected using an iBright 1500 Imaging System (Thermo Fisher Scientific, Waltham, MA, USA). Band intensity was quantified using the iBright analysis software (Thermo Fisher Scientific, version 5.2.0).

### 2.6. Quantitative RT-PCR (qRT-PCR)

Cell culture experiments were carried out as described previously. Total RNA was extracted from the cell lysates of RAW 264.7 using TRIzol reagent (Invitrogen, Waltham, MA, USA), following the manufacturer’s instructions. cDNA was synthesized using an All-in-one 5× cDNA master mix (Cellsafe, Yongin, Republic of Korea). The synthesized cDNA was mixed with the iQ SYBR Green Mastermix and pro-inflammatory cytokine-specific primers and analyzed by quantitative RT-PCR for 40 cycles of 95 °C for 15 s and 55 °C for 30 s. Gene expression was assessed using the 2^−ΔΔCt^ method (Quant3 studio system, Applied Biosystem, Waltham, MA, USA). The primer set of pro-inflammatory cytokines to analyze qRT-PCR is listed in Table 1.

### 2.7. RT^2^ Profiler PCR Array

Total RNA and cDNA were extracted from RAW 264.7, using the RT^2^ First Stand Kit (Qiagen, Hilden, Germany). The RT^2^ profiler ^TM^ PCR array mouse cytokines and chemokines (Qiagen, PAMM-150Z/330231) were analyzed according to the manufacturer’s instructions. The qPCR conditions were as follows: hold for 10 min at 95 °C followed by 40 cycles of 95 °C for 15 s and 60 °C for 60 s. The results were analyzed using GeneGlobe (https://dataanalysis2.qiagen.com/pcr, accessed on 8 February 2024), and the Ct values were normalized to the GAPDH internal housekeeping gene.

### 2.8. Statistical Analysis

Statistical data analysis was performed using GraphPad Prism 7.0 software (GraphPad Software, San Diego, CA, USA), and one-way ANOVA was used to identify group differences. The data are representative of at least three independent experiments. All experimental results are represented as the mean ± SD. *p*-values are shown as * *p* < 0.05; ** *p* < 0.01; *** *p* < 0.001; and **** *p* < 0.0001.

## 3. Results

### 3.1. Purification of Single Compounds from Seaweed Polysaccharides

**Figure 1:**
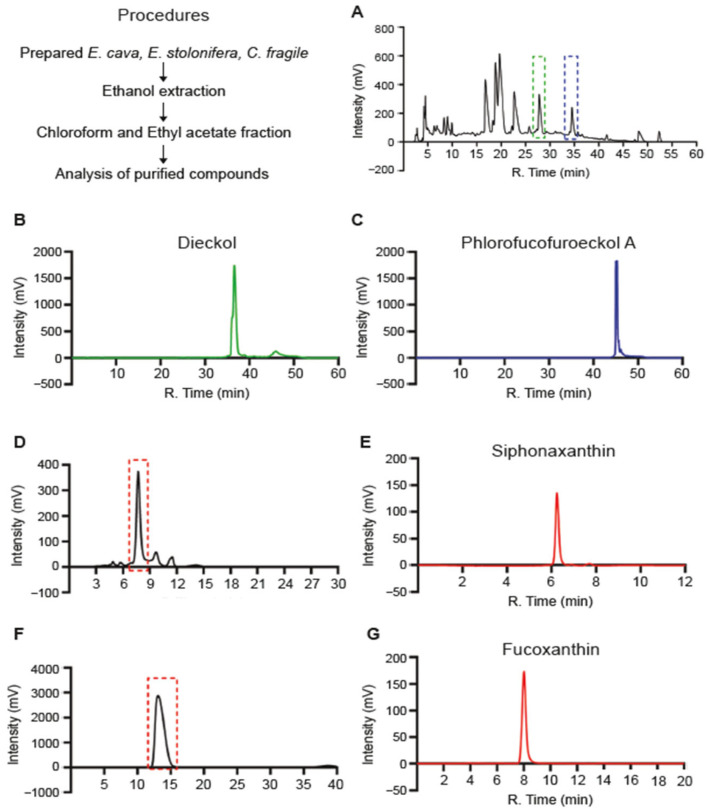
Purification of single compounds from seaweeds: (**A**) Preparative HPLC separation of (**A**) phlorotannin from the ethyl acetate fraction of *Ecklonia cava* (*E. cava*) and (**D**,**F**) chloroform fraction of *Ecklonia stolonifera* (*E. stolonifera*) and *Codium fragile* (*C. fragile*), respectively. HPLC chromatograms corresponding to purified from (**A**) to (**B**) Dieckol (green dashed), and (**C**) Phlorofucofuroeckol-A (blue dashed), (**E**) Siphonaxanthin, and (**G**) Fucoxanthin.

Ingredients with effective biofunctional properties differ depending on the type of seaweed used. First, we purified four single candidates from various seaweeds with potential anti-inflammatory properties. Fucoxanthin (Fx) and siphonaxanthin (Sx) were purified from *Ecklonia stolonifera* and *Codium fragile*, whereas phlorotannins, including dieckol (Dk) and phlorofucofuroeckol-A (PFF-A), were purified from *Ecklonia cava*. Briefly, dried seaweed was treated with ethanol and subjected to chloroform and ethyl acetate fractionation and further purification through high-performance liquid chromatography (HPLC) (Figure 1A,D,F). Purified Sx, Fx, Dk, and PFF-A peaks were observed at extract retention times of 6.3, 8, 38, and 45 min, respectively (Figure 1B,C,E,G). The four purified compounds extracted from seaweeds had equal retention times and purities, consistent with previous studies [21,22]. Structural determination of the phlorotannins, including Dk and PFF-A, was performed using ^1^H and ^13^C NMR (Figure S1). The LC-MS analyzed the purified Dk and PFF-A structures. Four and seven peaks, corresponding to each compound fragment, were detected (Figure S2). In the second MS analysis, all peaks yielded characteristic fragments derived from each compound. Based on the profile of the fragment ions, we concluded that the single compounds purified from seaweeds had accurate structures and were of high quality.

### 3.2. Cytotoxicity Analysis of Fine Dust and Single Compounds

**Figure 2:**
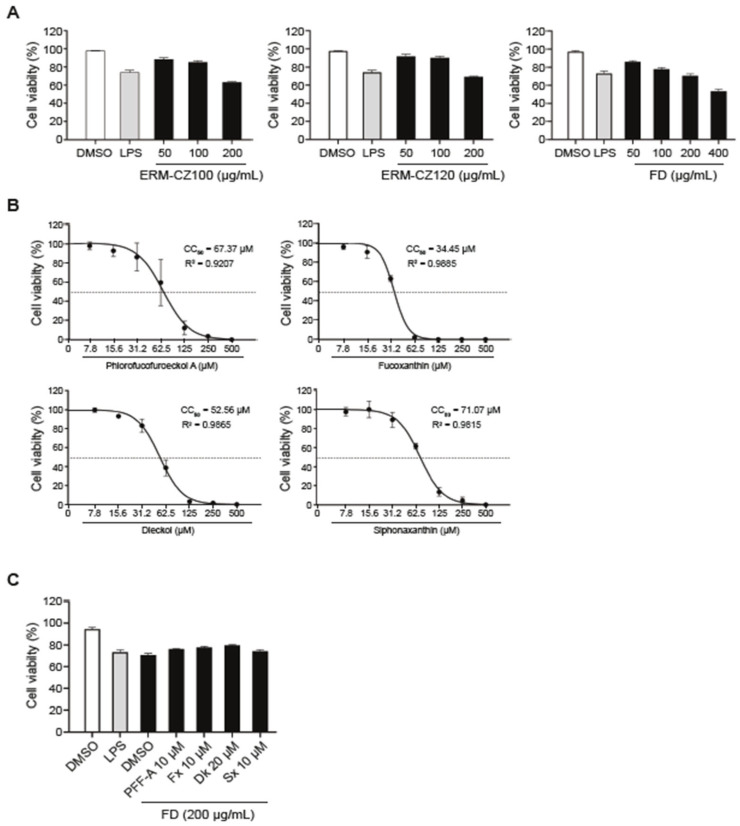
Cytotoxicity analysis of commercial FD and single compounds: (**A**) RAW 264.7 cells are treated with commercial fine dust ERM-CZ100 or ERM-CZ-120 or a mixture (FD) for 24 h at the indicated concentrations. The cell viability is assessed by live/dead cell staining using trypan blue and counting using a cell counter. (**B**) RAW 264.7 cells are treated with purified single compounds for 24 h at the indicated concentrations. The 50% cytotoxicity concentration (CC_50_) is calculated by a Cell Titer-Glo Luminescent Cell Viability Assay. Dashed line: cell viability of 50%. (**C**) RAW 264.7 cells are pre-treated with purified single compounds for 3 h at the indicated concentrations. The cells are treated with 1 µg/mL of LPS along with simultaneous FD stimulation. After 24 h, the cell viability is measured using the Cell Titer-Glo Luminescent Cell Viability Assay. Representative data are presented as the mean ± SD value.

Based on the hypothesis that single compounds exert anti-inflammatory effects, we determined the cytotoxicity of fine dust and single compounds in RAW 264.7 cells. The cytotoxicity of the fine dust was measured using a live/dead cell counter. Lipopolysaccharide (LPS) endotoxins are widely used to trigger inflammatory factors and were used as a standard to determine the cytotoxicity of fine dust. Two types of fine dust, ERM-CZ100 and ERM-CZ120, led to no toxicity at a concentration of less than 100 μg/mL, but, at a concentration of 400 μg/mL, cell viability was measured to be less than 55% after 24 h of incubation (Figure 2A). In addition, cell viability was over 70% in cells treated with a 200 μg/mL concentration by mixing two types of fine dust, termed FD (Figure 2A). Therefore, we performed subsequent studies with FD at a concentration of 200 μg/mL. Next, we measured the cytotoxicity of the individual compounds using a Cell Titer-Glo Luminescent Cell Viability Assay Kit (Promega).

The 50% cytotoxicity concentrations (CC_50_) of single compounds in cultured RAW 264.7 cells were determined to be 67.37 μM for PFF-A, 34.45 μM for Fx, 52.56 μM for Dk, and 71.07 μM for Sx at 24 h (Figure 2B). Furthermore, we measured the cytotoxicity of combinations of FD and single compounds. The RAW 264.7 cells were pre-treated with an appropriate concentration of each single compound for 3 h and then treated with 200 µg/mL of FD for 24 h. The results showed that the cell viability improved by 5–9% in cells treated with combinations with each compound compared with cells treated with FD alone (Figure 2C).

### 3.3. Single Compounds Inhibit FD-Induced Pro-Inflammatory Responses

**Figure 3:**
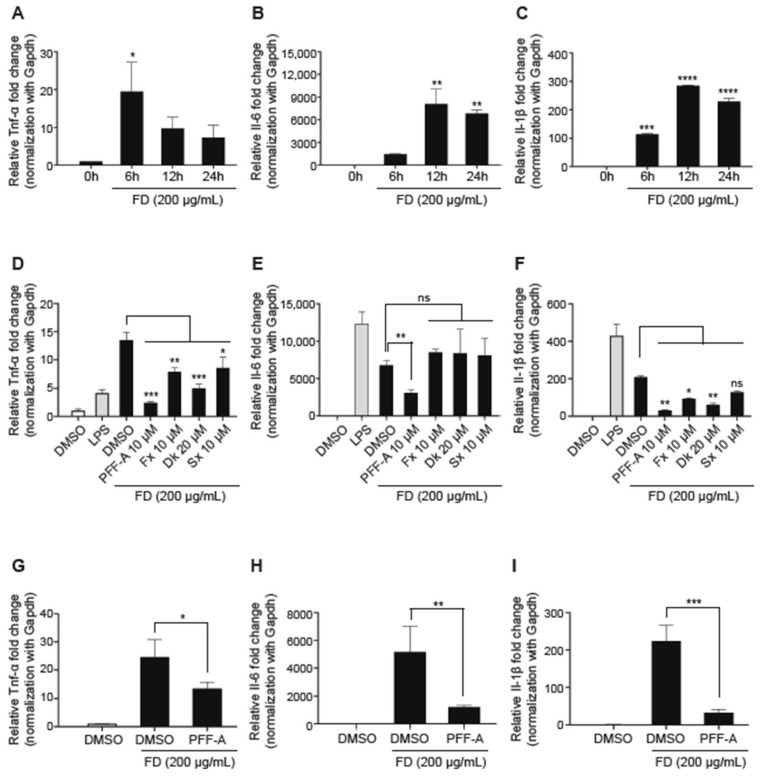
Effects of single compounds against FD-induced pro-inflammatory response: (**A**–**C**) RAW 264.7 cells are treated with 200 µg/mL of FD and the expression of pro-inflammatory cytokines was time-dependently measured by qRT-PCR. (**D**–**F**) RAW 264.7 cells are pre-treated with purified single compounds for 3 h at the indicated concentrations. After 3 h, the RAW 264.7 cells are stimulated using 1 µg/mL of LPS or 200 µg/mL of FD. The expression of pro-inflammatory cytokines is quantified using qRT-PCR after 24 h of incubation. (**G**–**I**) RAW 264.7 cells are pre-treated with 10 µM of PFF-A for 3 h. The expression of pro-inflammatory cytokines is quantified using qRT-PCR after 12 h of LPS or FD stimulation. Data are presented as the mean ± SD. One-way ANOVA with Tukey’s multiple-comparison test is performed to determine the significance (* *p* < 0.05, ** *p* < 0.01, *** *p* < 0.001, ns: not significant).

We investigated the expression of inflammatory cytokines to determine whether FD induces an inflammatory response in RAW 264.7 cells. First, we confirmed the inflammatory response according to dose-dependent FD through a preliminary study. The results in the expression levels of pro-inflammatory cytokines including tumor necrosis factor-alpha (Tnf-α), Interleukin 6 (Il-6), and Interleukin beta (Il-1β) were time-dependently regulated in RAW 264.7 cells treated with 200 μg/mL of FD (Figure 3A–C). The anti-inflammatory properties of single compounds were tested with 200 μg/mL of FD in RAW 264.7 cells. After pretreatment with a single compound for 3 h, the cells were washed with PBS and then incubated with FD for 24 h. The expression levels of FD-induced pro-inflammatory cytokines, including Tnf-ɑ and IL-1β, were significantly reduced by the four single compounds compared to FD only (Figure 3D,F). In contrast, the expression of Il-6 was not significant in RAW 264.7 cells pre-treated with Fx, Sx, and Dk, except for PFF-A (Figure 3E). Pro-inflammatory cytokines trigger early inflammatory responses. Therefore, we investigated the anti-inflammatory effects of PFF-A early in RAW264.7 cells treated with FD for 12 h. The 10 μM concentration of PFF-A effectively inhibited FD-induced inflammatory cytokines in RAW 264.7 cells treated with FD for 12 h (Figure 3G–I). These results indicate that a single compound, especially PFF-A, extracted from seaweeds has potent anti-inflammatory activity against the FD-induced inflammatory responses.

### 3.4. PFF-A Modulates NF-κB/MAPK-Mediated Il-1β Production

**Figure 4:**
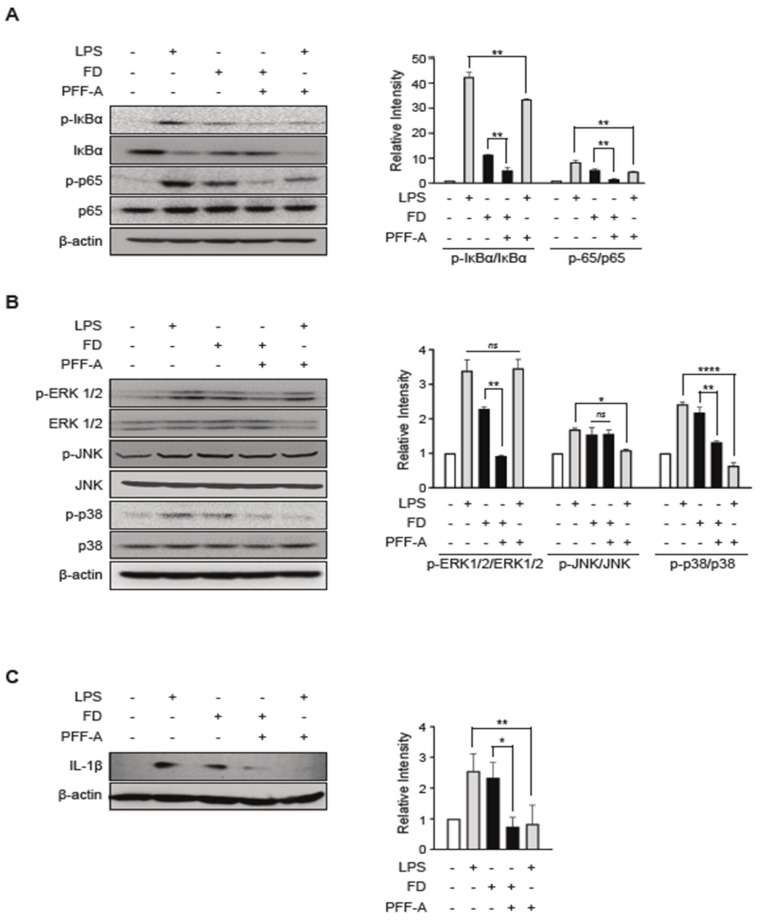
PFF-A exerts its anti-inflammatory effects by modulating the NF-κB/MAPK singling pathway: (**A**–**C**) RAW 264.7 cells are pre-treated with 10 µM of PFF-A for 3 h. The cells are harvested after 0.5 h (**A**) or 12 h (**B**,**C**) stimulated via LPS or FD and subjected to SDS-PAGE followed by Western blotting to determine the activation of inflammatory mediators, such as NF-κB, ERK1/2, JNK, and p38 MAPK and mature IL-1β production. The relative intensity of phosphorylation levels is normalized using the iBright analysis software (Thermo Fisher Scientific, Version 5.2.0). One-way ANOVA with a Tukey’s multiple-comparison test is performed to determine the significance (* *p* < 0.05, ** *p* < 0.01, **** *p* < 0.0001, ns: not significant).

Bioactive seaweed-derived polysaccharides have been reported to exhibit significant anti-inflammatory activity by modulating signaling pathways in several inflammatory models [23]. Hence, we investigated the mechanism by which PFF-A modulates anti-inflammatory responses. After pretreatment with PFF-A, the inflammatory response in RAW 264.7 cells was stimulated by LPS or FD. The results showed a significant decrease in the phosphorylated forms of IκBα and p65 levels in PFF-A pre-treated cells compared to non-treated cells (Figure 4A). Furthermore, pretreatment with PFF-A only partially rescued the phosphorylation of ERK and p38, but not that of JNK (Figure 4B).

Il-1β, a key regulator of inflammation, is well known to trigger a cascade of molecular pathways leading to the production of pro-inflammatory molecules [24]. The LPS or FD-induced Il-1 β production was markedly reduced by PFF-A pre-treatment (Figure 4C) in RAW 264.7 cells. These results indicated that PFF-A modulates FD-induced Il-1β production by regulating NF-kB and MAPK signaling.

### 3.5. PFF-A Plays an Anti-Inflammatory Role in FD-Induced Inflammatory Responses

**Figure 5:**
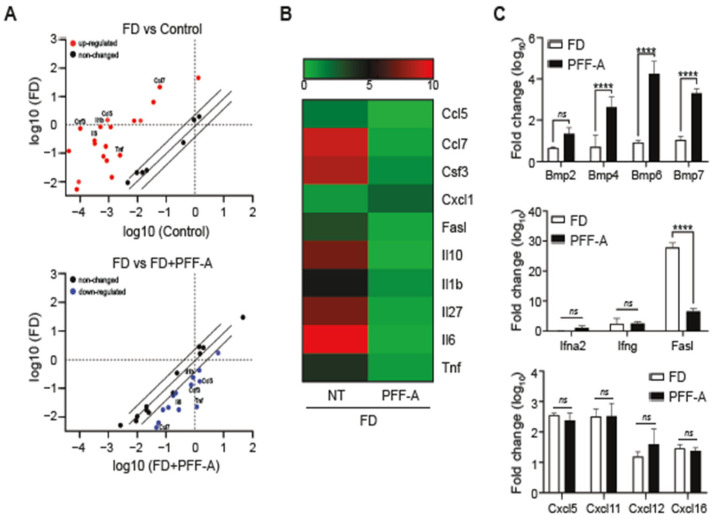
Profiling of inflammation-associated genes in FD-induced macrophage treated with PFF-A. cDNA extracted from FD-induced RAW 264.7 cells treated with/without PFF-A is analyzed simultaneously to profile 84 inflammatory genes by the RT2 profiler PCR assay: (**A**) The scatter plot shows the up-regulated (red), down-regulated (blue), and unchanged (black) genes. (**B**) The heat maps present the alteration of inflammatory gene expression. (**C**) The quantification of Bmp (metabologen), Fasl (apoptotic), Ifnα2 and Ifnγ (Type I interferon), and the C-X-C motif chemokine ligand (chemoattractant). Based on the GeneGlobe database (https://dataanalysis2.qiagen.com/pcr, accessed on 8 February 2024), the heat map for the RT^2^ profiler PCR assay is generated using GraphPad Prism 7.0 (https://www.graphpad.com/, accessed on 8 February 2024). Data are presented as the mean ± SD. Two-way ANOVA with Sidak’s multiple-comparison test is performed to determine the significance (**** *p* < 0.0001, ns: not significant).

We investigated the commitment of the pro-inflammatory cyto/chemokines in FD-exposed RAW 264.7 cells after PFF-A pre-treatment. The mouse inflammation RT^2^-profiler PCR array assessed the expression of inflammatory-related genes in RAW 264.7 cells exposed to FD with or without PFF-A pre-treatment. The scatter plot revealed that inflammation-related genes such as *Tnf-α*, *Il-6*, colony stimulating factor (*Csf3*), C-C chemokine ligand 5 (*Ccl5*), *Ccl7*, and *Il-1β*, which were up-regulated by FD stimulation, were down-regulated after PFF-A pre-treatment (Figure 5A). At 24 h, unsupervised clustering analysis illustrated that most inflammation-related genes were expressed at higher levels in RAW 264.7 cells, but these expression levels were rescued by PFF-A pretreatment (Figure 5B). Furthermore, the PFF-A pretreatment group showed upregulation of the bone morphogenetic protein (BMP) family, including Bmp2, Bmp4, Bmp6, and Bmp7. In contrast, the expression levels of interferon-related genes (Ifnα2 and Ifnγ) and CxC motif chemokine ligands (Cxcl5, Cxcl11, Cxcl12, and Cxcl16) did not differ between the groups (Figure 5C). These findings suggest that PFF-A improves cell survival by inhibiting early inflammatory responses.

## 4. Discussion

FD particles have become an important cause of air pollution, especially in East Asian countries such as China, the Republic of Korea, and Japan, and are considered to cause serious health problems [25]. In China and South Korea, air pollution caused by FD particles has emerged as a major environmental issue annually, and FD particles have been shown to be associated with various respiratory diseases [26,27,28,29]. The fine dust (PM10-like) ERM-CZ100 (organic constituents), and ERM-CZ120 (inorganic constituents)-induced inflammatory response in RAW264.7 cells have been reported [30]. Previous reports have described two possibilities by which PM induces an intracellular inflammatory response. The first explains that the source is the content of transition metal ions that influence inflammation through the oxidative stress pathway. Second, the bacteria-derived endotoxin bound to the particle surface triggers the inflammatory stimulus [31,32]. In this study, commercial FD (PM10-like) significantly increased the expression levels of pro-inflammatory genes such as *Tnf-α*, *Il-6*, and *Il-1β* in RAW 264.7 cells. Our data indicate that various organic components and trace elements in commercial FD promote inflammatory responses depending on the components adsorbed onto the particles, such as LPS stimulation [33]. Therefore, a risk of side effects was estimated when the components of commercial FD enter the air.

Diverse seaweeds in marine ecosystems are sources of bioactive compounds, polysaccharides, fatty acids, peptides, and proteins. The wide range of seaweed-derived bioactive compounds has valuable pharmaceutical potential, such as immunomodulation [34], anticancer properties [35], antioxidant potential, and anti-inflammatory activity [36,37,38]. Phlorotannins and carotenoids are natural bioactive compounds that are used as functional ingredients in food products owing to their various health benefits. Many previous studies have reported the benefits of phlorotannins and carotenoids in human health, including antioxidant, anti-inflammatory, anti-allergic, neuroprotective [39], and anticancer activities. Interestingly, increasing scientific evidence regarding their bioactivity has promoted their extraction for use in various fields, such as animal feed, functional foods, and the nutraceutical industry. Here, we extracted four single compounds from marine brown and green algae: phlorotannins, dieckol (Dk) and phlorofucofuroeckol-A (PFF-A), and carotenoids, such as siphonaxanthin (Sx) and fucoxanthin (Fx). The four purified natural compounds showed no toxicity at the concentrations tested (Figure 2). Among the four single compounds, PFF-A, one of the six phlorotannin classes, has been reported to successfully reduce LPS-induced nitric oxide (NO) production [40] and inhibit LPS-stimulated iNOS and COX-2 expression in LPS-stimulated macrophages [41]. Additionally, the inhibitory effects of PFF-A were associated with the halting of the ERK signaling pathway, as well as the inactivation of NF-κB signaling, which occurred because of the attenuation of IκB degradation, IκB phosphorylation, NF-κB translocation, and DNA binding in LPS-stimulated inflammation [42]. However, unlike LPS, the anti-inflammatory potential of PFF-A against FD-induced pro-inflammatory responses is poorly understood. Our results demonstrated that commercial FD significantly induced inflammatory cytokines such as Tnf-α, Il-6, and Il-1β, whereas treatment with the four purified single compounds, especially PFF-A, reduced FD-induced pro-inflammatory cytokines (Figure 3). Commercial FD markedly up-regulates mature IL-1β and the phosphorylation levels of NF-κB, ERK1/2, and p38 MAPK. In contrast, the NF-κB and MAPK pathways induced by FD through the inflammatory cytokine-mediated pathway were down-regulated by treatment with PFF-A, even in the LPS-stimulated inflammatory response (Figure 4). Based on these results, we hypothesized that FD induces intracellular inflammatory cytokines through TLR4/MyD88, similar to LPS, and that PFF-A inhibits pro-inflammatory responses by blocking upstream TLR4 signaling. Several studies reported that particulate matter (PM) in air induces cyclooxygenase (COX)-2 expression and reactive oxygen species (ROS) production, resulting in the regulation of cell fate through oxidative stress caused by mitochondrial dysfunction [42,43]. We expect PFF-A to control mitochondrial damage by modulating FD-induced inflammatory mediators, although further scientific studies are needed.

The cytokine analysis of FD-induced RAW 264.7, treated with or without PFF-A, revealed distinctly different inflammatory responses, whereas innate immunity and CXC motif chemokines were similar between the groups. Our results showed that FD induced the expression of pro-inflammatory genes including Il-6, Il-1, Tnf, Ccl5, Ccl7, and Csf3 compared to the control (Figure 5). In contrast, PFF-A treatment inhibited the FD-induced expression of pro-inflammatory and apoptotic genes, such as Fasl. The BMP family is known to play a pivotal role in the onset of inflammatory diseases [44]. Several studies have reported that inflammatory disorders have pathological features associated with changes in BMP expression and the BMP signaling pathway [45,46,47]. Interestingly, our results indicated that several BMP genes including Bmp2, Bmp4, Bmp6, and Bmp7 were markedly up-regulated following PFF-A treatment (Figure 5C). These results suggested that Bmp signaling may play a role in suppressing inflammatory states through the relationship with MAPK and NF-κB. Notably, from our gene expression analysis, we hypothesized that BMP genes may play a critical role in FD-induced inflammatory responses. Thus, further studies are required to address how PFF-A modulates pro-inflammatory mediators and prevents inflammation. Finally, through mechanistic studies of PFF-A metabolites, we suggest that PFF-A is a potential new therapeutic or preventive candidate for treating inflammatory diseases.

## 5. Conclusions

In this study, we purified four single potential seaweed anti-inflammatory bioactive compounds, including Sx, Fx, Dk, and PFF-A, that can prevent FD-induced inflammatory responses. Structural analysis was performed on all compounds through NMR and LC/MS. Among the four compounds, PFF-A markedly prevented inflammatory cytokines, including Tnf-α, Il-6, and Il-1β in RAW 264.7 cells without cytotoxicity. Moreover, PFF-A not only inhibited IL-1β expression by regulation of the NF-κB/MAPK signaling pathway but also reduced the expression levels of pro-inflammatory and apoptosis-related genes in RAW 264.7 cells. These findings suggest that PFF-A may be a potential therapeutic candidate for inflammatory responses induced by fine dust. However, nonclinical studies, pharmacokinetic analyses, and clarification of PFF-A metabolites are needed to develop therapeutics for systemic inflammation.

## Figures and Tables

**Table 1:** Primer sequences for qRT-PCR analysis.

Genes	Forward (5′-3′)	Reverse (5′-3′)
Tnf-α	CCCCAAAGGGATGAGAAGTT	CACTTGGTGGTTTGCTACGA
Il-6	CCGGAGAGGAGACTTCACAG	CAGAATTGCCATTGCACAAC
Il-1β	GGATGAGGACATGAGCACCT	AGCTCATATGGGTCCGACAG
Gapdh	CATCACTGCCACCCAGAAGACTG	ATGCCAGTGAGCTTCCCGTTCAG

Tnf-α, tumor necrosis factor-α; Il-, Interleukin-; and Gapdh, glyceraldehyde 3-phosphate dehydrogenase.

## Data Availability

The data supporting the findings of this study are available from the corresponding author upon reasonable request.

## Supplementary Materials

The following supporting information can be downloaded at https://www.mdpi.com/article/10.3390/medicina61010165/s1. All supplementary data are included in the Supplementary Materials, Figure S1: NMR spectra of Dk and PFF-A; and Figure S2: LC-MS/MS spectra of structural fragments of Dk and PFF-A.
