# Supplementary material for: Phlorofucofuroeckol-A: A Natural Compound with Potential to Attenuate Inflammatory Diseases Caused by Airborne Fine Dust
Source: Medicina (Kaunas). 2025 Jan 20;61(1):165. doi: 10.3390/medicina61010165 (PMC11767036; doi:10.3390/medicina61010165)
Supplement: Supplementary file 1 [file medicina-61-00165-s001.zip › Supplementary Materials-.pdf]

Supplementary figure legend

Supplementary Figure 1. Analysis of the NMR spectrum for a Dk and PFF-A. (A) Dk; ^{1}H-NMR (MeOH-d_4, 400 MHz): δ 6.16 (1H, s, H-3"), 6.14 (1H, s, H-3), 6.10 (1H, s, H-2''', H-6'''), 6.07 (1H, d, J = 2.8 Hz, H-6), 6.06 (1H, d, J = 2.8 Hz, H-8), 5.99 (1H, d, J = 2.8 Hz, H-8"), 5.96 (1H, d, J = 2.8 Hz, H-6"), 5.94 (1H, s, H-2', H-4', H-6'); ^{13}C NMR (MeOH-d_4, 100 MHz): δ 161.8 (C-1'), 160.1 (C-3', C-5'), 157.7 (C-1'''), 155.9 (C-7), 154.5 (C-7"), 152.3 (C-3''', C-5'''), 147.3 (C-2), 147.2 (C-2"), 147.1 (C-9"), 146.8 (C-9), 144.3 (C-5a"), 144.1 (C-4"), 143.4 (C-4), 143.3 (C-5a), 138.6 (C-10a"), 138.4 (C-10a), 126.4 (C-4'''), 126.1 (C-9a), 125.6 (C-4a), 125.5 (C-4a"), 124.8 (C-9a"), 124.6 (C-1"), 124.5 (C-1), 99.9 (C-8"), 99.7 (C-8), 99.5 (C-3"), 99.4 (C-3), 97.6 (C-4'), 96.2 (C-2''', C-6'''), 95.8 (C-6), 95.7 (C-6"), 95.3 (C-2', C-6'). (B) PFF-A; ^{1}H-NMR (MeOH-d_4, 400 MHz): δ 6.64 (1H, s, H-13), 6.42 (1H, s, H-9), 6.27 (1H, s, H-3), 5.97 (2H, d, J = 2.1 Hz, H-4', H-4"), 5.95 (1H, d, J = 2.0 Hz, H-2'), 5.93 (1H, t, J = 2.0 Hz, H-2"), 5.90 (2H, d, J = 2.1 Hz, H-6', H-6"); ^{13}C NMR (MeOH-d_4, 100 MHz): δ 161.9 (C-1'), 161.8 (C-1"), 160.2 (C-3", C-5"), 160.1 (C-3', C-5'), 153.2 (C-12a), 151.7 (C-10), 151.1 (C-11a), 148.3 (C-2), 148.2 (C-8), 145.9 (C-14), 143.9 (C-4), 138.4 (C-15a), 135.3 (C-5a), 128.0 (C-14a), 124.9 (C-1), 124.7 (C-4a), 122.3 (C-11), 105.3 (C-7), 105.2 (C-6), 99.9 (C-9), 99.3 (C-3), 97.7 (C-4), 97.6 (C-4"), 96.2 (C-13), 95.4 (C-2', C-6'), 95.3 (C-2", C-6").

Supplementary Figure 2. Analysis of LC-MS/MS spectrum for structural fragment of Dk and PFF-A. The Orbitrap Exploris 120 mass spectrometer analyzed the single compounds to detect structural fragments of each compound. MS/MS analysis of fragments; Dk, ESI-HRMS *m/z* 741.0692 $[M - H]^-$ and PFF-A, ESI-HRMS *m/z* 601.0952 $[M - H]^-$ indicated.
