# Supplementary figures and images for: Phlorofucofuroeckol-A: A Natural Compound with Potential to Attenuate Inflammatory Diseases Caused by Airborne Fine Dust
Source: Medicina (Kaunas). 2025 Jan 20;61(1):165. doi: 10.3390/medicina61010165 (PMC11767036; doi:10.3390/medicina61010165)

Supplementary Figure 1

A

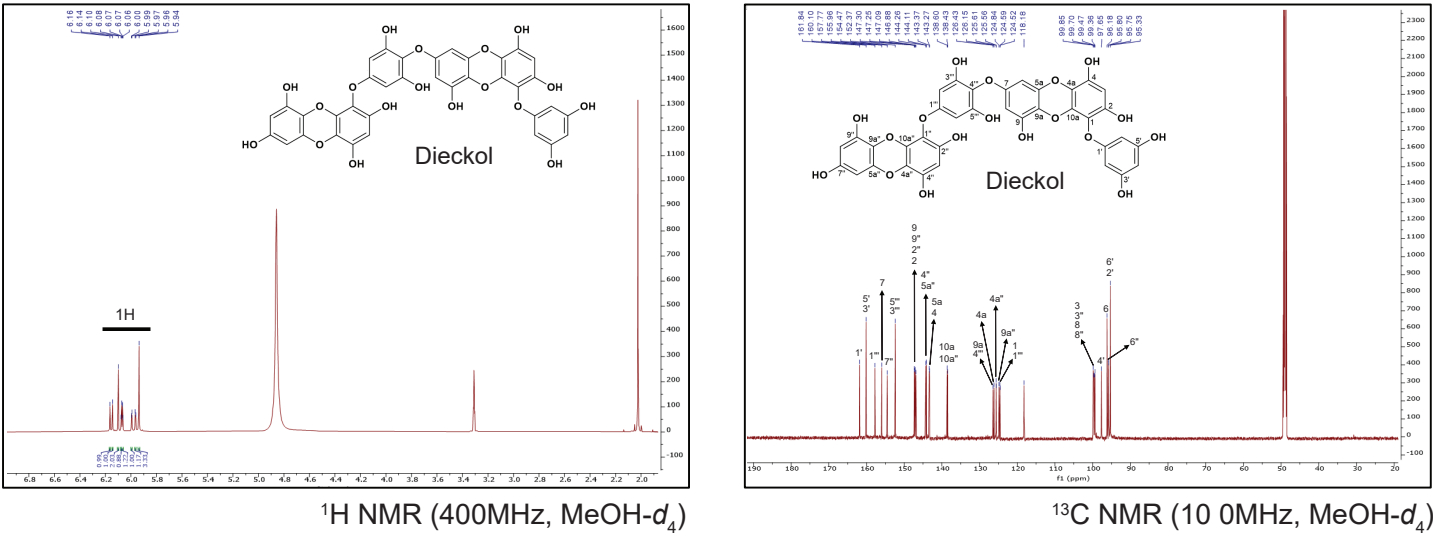

B

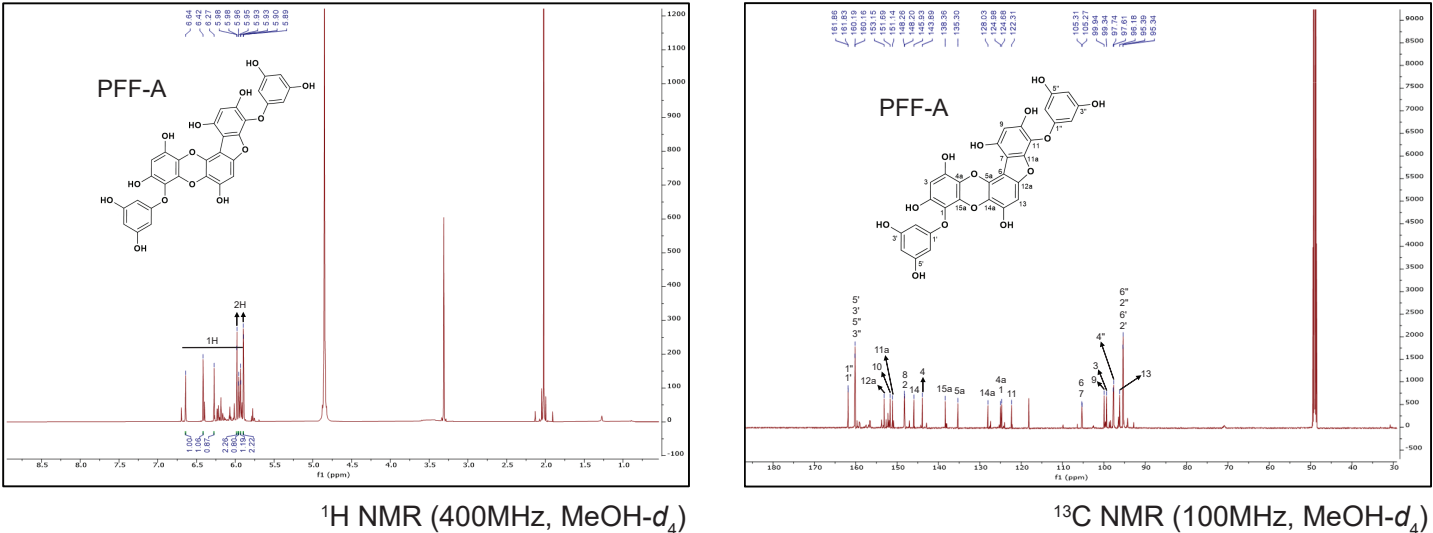

# Supplementary Figure 2

A

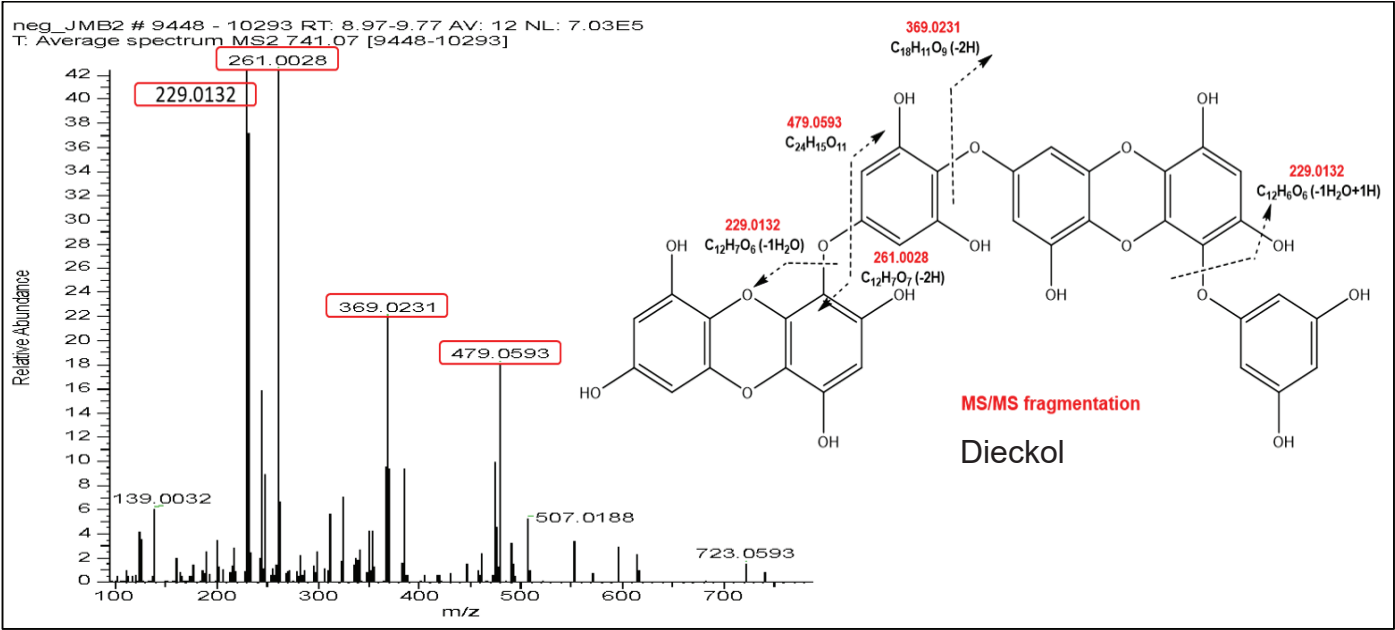

B

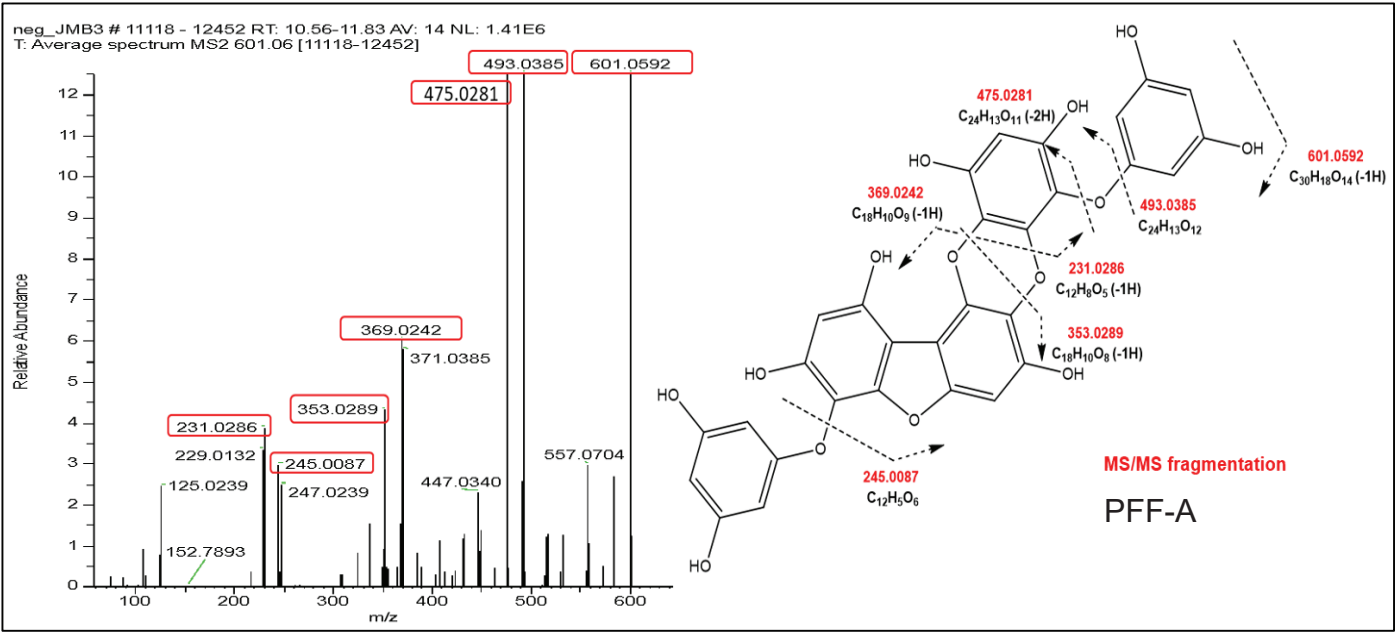

Supplement: Supplementary file 1 [file medicina-61-00165-s001.zip › Supplementary Materials.pdf]
